# Treatment of Alcohol Dependence With Drug Antagonists of the Stress Response

**DOI:** 10.35946/arcr.v34.4.15

**Published:** 2012

**Authors:** Amanda E. Higley, George F. Koob, Barbara J. Mason

**Affiliations:** **Amanda E. Higley, Ph.D.,***is a postdoctoral research fellow at the Committee on the Neurobiology of Addictive Disorders, The Pearson Center for Alcoholism and Addiction Research, The Scripps Research Institute, La Jolla, California.*; **George F. Koob, Ph.D.,***is a professor at the Committee on the Neurobiology of Addictive Disorders, The Pearson Center for Alcoholism and Addiction Research, The Scripps Research Institute, La Jolla, California.*; **Barbara J. Mason, Ph.D.,***is a professor at the Committee on the Neurobiology of Addictive Disorders, The Pearson Center for Alcoholism and Addiction Research, The Scripps Research Institute, La Jolla, California.*

**Keywords:** Alcohol dependence, alcoholism, alcohol use disorder, stress, stress response, relapse, relapse prevention, antagonists, negative affective states, treatment, neuroadaptation, pharmacotherapy, brain, neurochemistry

## Abstract

Alcohol dependence is a chronic relapsing disorder characterized by neuroadaptations that may result in the emergence of negative affective states and stress responses upon discontinuation of alcohol use. Clinical studies have demonstrated that alcohol-dependent people are more sensitive to relapse provoking cues such as alcohol, negative affect, and stress. Moreover, stress relief during protracted abstinence is thought to be a major motivation for excessive alcohol consumption. The relationship between chronic alcohol use, stress, and relapse has implications for the treatment of alcohol dependence. Recent research suggests that neural systems mediating stress responses may offer useful targets for pharmacotherapy of alcoholism.

Although alcohol dependence affects 4 percent of the adult population and is the third leading cause of preventable death in the United States (Substance Abuse and Mental Health Services Administration 2009), fewer than 15 percent of people with alcoholism receive treatment (Hasin et al. 2007). The *Diagnostic and Statistical Manual of Mental Disorders–Fourth Edition Text Revision (DSM–IV–TR)* (American Psychiatric Association 2000) characterizes alcohol dependence as a maladaptive pattern of drinking leading to clinically significant impairment, as manifested by a compulsion to drink, a lack of control over the amount of alcohol consumed, and continued drinking despite realization of the associated problems. Despite significant progress in the development of efficacious behavioral and pharmacologic treatments for alcohol dependence, relapse rates remain very high. Relapse is one of the principle characteristics of alcohol dependence. Given that one of the most challenging aspects of recovering from alcohol dependence is maintaining abstinence, understanding the factors underlying relapse susceptibility is especially important. Research indicates that alcohol-associated cues, negative-affective states, and stress are common relapse triggers (Higley et al. 2011; Mason et al. 2008; Sinha et al. 2009).

Several neurochemical systems and brain regions are involved in the development of alcohol dependence (for review, see Koob and Le Moal 1997). Such neuroadaptations may result in the emergence of negative-affective states and stress responses upon discontinuation of alcohol use, thus motivating dependent people to resume drinking. Alcohol is a powerful activator of the stress response. Chronic alcohol use is associated with several atypical stress responses, which could have important implications for understanding the neurobiology of dependence and relapse. Specifically, alcohol-dependent individuals show decreased release of the stress hormones cortisol and adrenocorticotropic hormone (ACTH) in response to acute intervening stressors (Berman et al. 1990; Wand and Dobs 1991), an effect that remains for up to 12 weeks after cessation of drinking (Bernardy et al. 1996; Ehrenreich et al. 1997; Errico et al. 1993; Lovallo et al. 2000). These attenuated reactions of the hypothalamic–pituitary–adrenal (HPA) axis, which controls the body’s major hormonal stress response, have been associated with alcohol relapse (Junghanns et al. 2003) and suggest that neural systems mediating stress responses may offer useful targets for pharmacotherapy of alcoholism.

Stress relief during protracted abstinence is thought to be a major motivation for excessive alcohol consumption. The signaling molecule corticotropin-releasing factor (CRF), a 41–amino acid neuropeptide^1^ with wide distribution throughout the brain and high concentrations in cell bodies in part of the hypothalamus (i.e., the paraventricular nucleus), the group of structures located near the bottom of the front of the brain (i.e., the basal forebrain), and notably the extended amygdala^2^ and brainstem, has been shown to play an integral role in mediating behavioral stress responses (Funk et al. 2006; Merlo Pich et al. 1995; Olive et al. 2002). CRF produced in and released from the hypothalamus activates the HPA axis. The physiologic mechanism of stress relief following alcohol consumption is thought to occur mainly in the extended amygdala outside the HPA system (for review, see Heinrichs and Koob 2004). However, the HPA axis may contribute to the dysregulation of the extended amygdala stress system. Acute alcohol administration has been shown to enhance levels of HPA axis hormones in humans and animal models (for review, see Koob and Le Moal 1997; Koob 2003). As dependence on alcohol develops, the extended amygdala stress system becomes sensitized and HPA axis activity appears to become dysregulated, and over time, chronic exposure to alcohol may actually decrease the responsiveness of the HPA axis to external stimuli, potentially impairing a person’s ability to cope with relapse-inducing stressors (Junghanns et al. 2003; Le et al. 2000; Zorrilla et al. 2001; see above).

Such alcohol-induced neurobiological changes represent possible molecular targets for pharmacotherapies of alcoholism, which help to facilitate abstinence or greatly reduce alcohol consumption by stabilizing neurobiological systems dysregulated by chronic alcohol use. Medications that normalize the dysregulation or balance of the reward and stress systems may protect against relapse. In fact, evidence shows that pharmacological treatments can support abstinence or decrease the number of heavy drinking days. Three medications are approved for the treatment of alcohol dependence in the United States––disulfiram, naltrexone, and acamprosate. Recent efforts to develop new medications have focused on specific neural responses to factors (e.g., stress) that increase risk of relapse to heavy drinking during protracted abstinence. The following sections will describe a series of neuropharmacological agents that alter the stress response and have potential for or have been used in the treatment of alcohol dependence.

## CRF Antagonists

Recent research has led to the hypothesis that the transition to alcohol dependence involves the dysregulation not only of neural circuits involved in reward but also of circuits that mediate behavioral responses to stressors. Alcohol-induced dysregulation of the brain’s stress and anti-stress systems is hypothesized to contribute to the negative emotional state characteristic of alcohol withdrawal. More specifically, several observations indicate that CRF contributes to the development of alcohol dependence. For example, alcohol is a powerful activator of stress systems involving both the HPA axis and extrahypothalamic CRF systems in the extended amygdala; the latter also become hyperactive during withdrawal, leading to increased CRF levels in certain brain regions (i.e., the central nucleus of the amygdala [CeA] and the BNST) (Funk et al. 2006; Merlo Pich et al. 1995; Olive et al. 2002). In animal models, acute withdrawal and protracted abstinence from alcohol and all other major drugs of abuse produce anxiety-like responses that are mediated by CRF and can be reversed by agents that block or reverse the actions of CRF (i.e., CRF receptor antagonists) (for review, see Heilig and Koob 2007). Preclinical studies show that CRF antagonists block alcohol withdrawal–induced anxiety (Baldwin et al. 1991), and CRF may be involved in increased alcohol self-administration during withdrawal (Valdez et al. 2002). Likewise, injections of small molecule antagonists of the CRF-1 receptor blocked increased alcohol intake during acute withdrawal and protracted abstinence in alcohol-dependent rats (Funk and Koob 2007). Moreover, CRF antagonists reduce stress-induced reinstatement to alcohol seeking (Le et al. 2000; Liu and Weiss 2002).

Dysregulation of the brain CRF system (innate or resulting as a maladaptive response to drugs of abuse or stress) seems to be one of the major elements common to depression, anxiety, and addiction. Genetic studies indicate an association between polymorphisms of the *CRHR1* gene and drinking behavior. Treutlein and colleagues (2006) found a significant correlation between *CRHR1* gene polymorphisms and both binge drinking and lifetime prevalence of alcohol intake in an adolescent sample from the Mannheim Study of Children at Risk^3^ as well as years of heavy drinking in a sample of adult alcoholics (Treutlein et al. 2006). Polymorphisms in the *CRHR1* gene also were found to moderate the relationship between the number of negative life events and rates of lifetime alcohol use and excessive alcohol use per occasion in the same study sample (Blomeyer et al. 2008), suggesting a clinical relevance for the CRF system in the treatment of alcoholism.

The above evidence suggests that the CRF system may be implicated in stress-induced relapse to alcohol drinking and that CRF antagonists may have therapeutic potential in alcohol dependence, particularly for people with genetic variants in the *CRHR1* gene that exacerbate a stress-induced susceptibility to alcohol dependence and relapse (Clinicaltrials.gov NCT01187511, 2010, Clinicaltrials.gov NCT01227980, 2011).

## α1-Noradrenergic System

Advances in the understanding of the neurobiology of alcohol dependence and relapse offer preclinical evidence that the noradrenergic systems (i.e., those related to the stress hormone and neurotransmitter norepinephrine) have intimate involvement in brain processes relevant to alcohol dependence and contribute to the brain stress activation associated with withdrawal. A study of recently abstinent alcohol-dependent patients revealed elevated plasma levels of norepinephrine and the related neurotransmitter epinephrine (Ehrenreich et al. 1997), suggesting central noradrenergic overdrive may play an important role in alcohol dependence. Moreover, the use of pharmacological ligands targeting both pre- and postsynaptic noradrenergic receptor subtypes attenuates certain symptoms of alcohol withdrawal (Riihioja et al. 1997).

Prazosin, an α1-noradrenergic receptor antagonist, has kindled interest as an effective drug in reducing alcohol use. Pfizer Pharmaceuticals introduced Prazosin in 1973 as an antihypertensive drug. An inexpensive generic drug for many years, prazosin has been used chronically by millions of people for hypertension. It is the most lipid soluble α1-noradrenergic antagonist and the only clinically available α1-noradrenergic antagonist demonstrated to be active at central nervous system sites when administered peripherally (Menkes et al. 1981). Prazosin blocks the α1-noradrenergic receptor implicated in stress responsivity and possibly in driving forebrain CRF release. Prazosin reduced self-administration of alcohol in both dependent and nondependent rats during acute withdrawal. However, prazosin was more potent in dependent animals, suggesting an increase in the sensitivity to Prazosin in dependent animals due to alterations in the norepinephrine system during chronic exposure to alcohol (Walker et al. 2008). Rasmussen and colleagues (2009) demonstrated the efficacy of acute and chronic Prazosin treatment in suppressing alcohol drinking in rats selectively bred for alcohol preference.

A 6-week, double-blind, placebo-controlled pilot study of Prazosin for the treatment of alcohol dependence reported a significant reduction in drinking behavior in actively drinking alcohol dependent patients (Simpson et al. 2009). Large controlled studies currently are in progress to further investigate the role of Prazosin in alcohol dependence (e.g. NCT00762710, 2010).

## Neurokinin 1 (NK1) Receptor and Substance P Antagonists

Targeting the receptor system for Substance P, which modulates emotional states, has been suggested as a viable therapeutic target for the treatment of alcohol dependence (Ebner et al., 2009). Substance P, a neurotransmitter from the tachykinin family, is released in response to stress, and preferentially binds to the NK1 receptors, which are highly expressed in brain regions critical for the regulation of emotional behavior and neurochemical responses to stress (for review see Commons 2010). Substance P also facilitates stress-induced HPA axis activation as reflected in ACTH and cortisol levels (for review see Ebner and Singewald 2006). Noxious or aversive stimuli activate Substance P pathways. In addition, Substance P administration into the brain produces anxiety-inducing and aversive effects (Aguiar and Brandao 1996, Elliott 1988, Teixeira et al.1996). Furthermore, mice that lack the NK1 receptor have been found to consume lower quantities of alcohol compared with control animals (for review see George et al. 2008).

A double-blind clinical trial of alcohol dependence found treatment with an NK1 antagonist significantly decreased craving, blunted cortisol responses, and decreased functional magnetic resonance imaging responses to affective stimuli in recently detoxified alcohol-dependent study participants (for review, see George et al. 2008). Together, these results suggest that Substance P-NK1 systems may play a role in drug reward, dependence, and reinstatement.

## Neuropeptide Y

Neuropeptide Y (NPY), a 36–amino acid peptide, also is involved in regulating the body’s stress response but with a neural and behavioral profile that in almost every aspect is opposite to that of CRF. For example, NPY has powerful anxiety-reducing effects in animals. It is one of the most abundant neuropeptides in the central nervous system (CNS) and is considered an important regulating factor in emotional behavior. Administration of NPY from an external source (i.e., exogenous NPY) has antianxiety and sedative effects that rely, at least partially, on activation of Y_1_, a G-protein–coupled receptor located in the amygdala (Britton et al. 1997; Broqua et al. 1995; Heilig et al. 1993; Heilig and Thorsell 2002).

Several findings point to a role for NPY produced in the body (i.e., endogenous NPY) in the control of stress- and anxiety-related behaviors, supporting the antistress effects observed following central administration of NPY. In animal models, acute physical restraint, which promotes experimental anxiety, suppresses NPY expression within the amygdala and cortex, an effect that parallels the anxiety-inducing effects of stress. In contrast, repeated exposure to a siren stressor leads to complete behavioral and endocrine habituation, accompanied by an upregulation of amygdalar NPY expression (Thorsell et al. 1999, 2010). These findings suggest that NPY expression seems to be involved in the behavioral adaptation to stressors.

NPY levels are lower in the CeA of alcohol-preferring (P) rats compared to non-P (NP) rats, and NPY infusion in the CeA attenuates the anxiety-like and alcohol drinking behaviors of P rats. Thus, a deficiency in NPY signaling in the CeA may be involved in regulating both anxiety and alcohol-drinking behaviors (Zhang et al. 2010) and NPY system modifications can influence alcohol intake (Ehlers et al. 1998; Hwang et al. 2004; Hwang et al. 1999). Furthermore, stimulation of NPY activity in this brain structure suppresses anxiety-like behavior (for review, see Thorsell 2007) and dependence-induced increases in alcohol drinking (Gilpin et al. 2008). Administration of NPY into the cerebral ventricles of the brain (i.e., intracerebroventricular infusion) in rats dose-dependently blocks the reinstatement of alcohol-seeking induced by a pharmacological stressor (Cippitelli et al. 2010). Moreover, alcohol-dependent rats exhibit decreased NPY content in the CeA during withdrawal (Roy and Pandey 2002), whereas, as stated above, CRF levels in this brain region are increased in alcohol-dependent animals. Together, these preclinical studies suggest that the NPY receptor may represent a novel pharmacological target for alcoholism.

## Dynorphin/κ Opioid System

Dynorphins are opioid peptides that derive from the prodynorphin precursor and are the presumed endogenous ligands for the κ opioid receptor (Chavkin et al., 1982). Dynorphins have widespread distribution in the CNS and play a role in a wide variety of physiological systems, including neuroendocrine regulation, pain regulation, motor activity, cardiovascular function, respiration, temperature regulation, feeding behavior, and stress responsivity (Koob 2008). Products of prodynorphin processing include dynorphin A(1–17), dynorphin A(1–8), and dynorphin B(1–29). Immunocytochemical distribution of dynorphin A and B shows significant cell bodies and terminals in addiction-relevant brain areas, such as the nucleus accumbens, CeA, BNST, and hypothalamus (Koob 2008).

Activation of the dynorphin/κ receptor system can produce analgesic actions similar to other opioids but also actions that are opposite to those of μ opioid receptors in the motivational domain, where dynorphins produce aversive, dysphoric-like effects in animals and humans (Shippenberg et al. 2007). Dynorphin has long been hypothesized to mediate negative emotional states. κ receptor agonists produce place aversions in rodents (Mucha and Herz 1985) and depression and dysphoria in humans (Pfeiffer et al. 1986). κ agonists also increase brain stimulation reward thresholds (Todtenkopf et al. 2004). Dynorphin inhibits dopamine release, both via the origins and terminals of the mesolimbic dopamine system, and this effect has been hypothesized to contribute to the aversive effects of dynorphin (Spanagel et al. 1992).

The evidence for a role of the dynorphin/κ opioid system in the neuroadaptive actions of ethanol (i.e., alcohol) is based both on biochemical studies and antagonist studies. Chronic self-administration of ethanol in C57BL/6J mice produced increases in dynorphin B in the amygdala and substantia nigra 21 days after cessation of drinking (Ploj et al. 2000). Chronic ethanol produced a decrease in κ opioid receptors in the nucleus accumbens (Rosin et al. 1999) and an increase in dynorphin B expression in the nucleus accumbens (Lindholm et al. 2000), providing further evidence of upregulation of dynorphin systems with ethanol dependence. Direct support for the hypothesis that dynorphin is part of the negative emotional systems recruited in dependence is the observation that a κ antagonist, norbinaltorphimine (nor-BNI), when injected intracerebroventricularly or systemically, blocked ethanol self-administration in dependent, but not in nondependent, animals (Doyon et al. 2006; Walker and Koob 2008; Walker et al. 2010). κ knockout mice also drank less ethanol in a two-bottle choice test using escalating doses of ethanol (Kovacs et al., 2005).

Stress also increases dynorphin activity (Shirayama et al. 2004), suggesting a potential interaction with CRF systems. Forced swim stress and inescapable footshock produced place aversions in mice that were blocked by a κ antagonist and dynorphin knockout. In other studies, CRF was hypothesized to produce its aversive effect via dynorphin activation (Land et al. 2008). Evidence also exists showing that reinstatement of drug-seeking behavior via activation of κ opioid receptors is mediated by CRF (Valdez et al. 2007). Thus, the dynorphin/κ system mimics stressor administration in animals in producing aversive effects and inducing drug-seeking behavior, and this aversive response may involve reciprocal interactions with nucleus accumbens dopamine and the brain extrahypothalamic CRF system. Thus, the dynorphin/kappa peptide system may be a parallel extrahypothalamic brain stress system that interfaces between the loss of reward function and gain in brain stress function associated with the transition to alcohol dependence (Koob et al. 2008).

## Summary

Alcohol has a complex neuropharmacology and can affect many different neurotransmitter systems. Several pharmacological agents that interact with specific neurotransmitter systems affected by alcohol already have shown efficacy in the treatment of alcohol dependence and many exciting experimental agents are on the horizon. Stress relief during protracted abstinence is thought to be a major motivation for excessive alcohol consumption and the present overview outlines several new targets for medications development based on interactions with the brain stress systems. The development of these agents has been based on translational approaches ranging from the use of molecular techniques to understand alcohol neurobiology and identify candidate molecules, to the use of numerous animal models of alcohol-related behaviors to test the effects and mechanisms of action underlying these agents, and finally the use of human clinical trials and laboratory paradigms to evaluate the clinical efficacy of these agents. Future research needs to focus on realizing the therapeutic potential of agents acting on the brain stress systems and examining genetic and patient-specific predictors of treatment response. A better understanding of the mechanisms underlying treatment response could lead to appropriate treatment matching and efficient utilization of such novel medications.

## Footnotes

1 For definitions of this term and other technical terms used in this article, see the Glossary on page 522–524.

2 The amygdala is an almond-shaped group of neurons located deep within the medial temporal lobe of the brain. They encompass several nuclei, or structures in the central nervous system, including the central, lateral, and basal nuclei. The extended amygdala is hypothesized to be a group of structures that includes the central nucleus of the amygdala, bed nucleus of the stria terminalis (BNST), and a transition zone in the shell of the nucleus accumbens.

3 The Mannheim Study of Children at Risk is a longitudinal study that followed children over a period of more than 20 years from infancy to adulthood.
